# Ideal spectral emissivity for radiative cooling of earthbound objects

**DOI:** 10.1038/s41598-020-70105-y

**Published:** 2020-08-03

**Authors:** Suwan Jeon, Jonghwa Shin

**Affiliations:** Department of Materials Science and Engineering, Korea Advanced Institute of Science and Technology, Daejeon, 34141 Republic of Korea

**Keywords:** Atmospheric optics, Mid-infrared photonics, Green photonics, Thermoelectric devices and materials

## Abstract

We investigate the fundamental limit of radiative cooling of objects on the Earth's surfaces under general conditions including nonradiative heat transfer. We deduce the lowest steady-state temperature attainable and highest net radiative cooling power density available as a function of temperature. We present the exact spectral emissivity that can reach such limiting values, and show that the previously used 8–13 μm atmospheric window is highly inappropriate in low-temperature cases. The critical need for materials with simultaneously optimized optical and thermal properties is also identified. These results provide a reference against which radiative coolers can be benchmarked.

## Introduction

The atmosphere acts as a selective window to electromagnetic waves, transmitting most of the visible sunlight and blocking harmful ultraviolet and X-rays^1^. Recently, the transparency window in the long-wavelength infrared, which ranges from 8 to 13 μm, has received great attention because it allows for below-ambient cooling without energy consumption^2–15^. The principle of this spontaneous cooling is based on thermal radiation in the transparency window being able to transfer heat between an object on the Earth’s surface and the cold outer space without being blocked by the atmosphere. This in turn relies on the fact that the outer space appears "cold" and reflects the young, finite-sized, and expanding nature of our universe. Even under direct sunlight, below-ambient cooling has been experimentally achieved by enhancing the reflectivity in the solar spectrum and the emissivity in the transparency window using various methods^3,7,9–12,15^. However, it remains unproven whether the 8.0–13.0 μm range is the exact optimal range to realize the maximum temperature drop versus ambient temperature, and the fundamental limit of the achievable cooling temperature has not been theoretically derived yet.

Here, we present the ideal spectral emissivity under general conditions that realizes the ultimate lower bound of the temperature of a radiatively cooled object on earth. Contrary to common belief, we prove that the optimal window is different from 8 to 13 μm and depends strongly on the target temperature, which in turn is limited by the non-radiative heat transfer rate. Particularly, with no non-radiative heat transfer, the cooler should have a needle-like spectral emissivity if the objective is to have as low steady-state temperature as possible, and can induce 56.4 K and 92.5 K drops in summer and winter, respectively, if enough time is given to reach a steady state. More generally, we establish a universal guideline for designing spectral emissivity under given environmental conditions, including ambient temperature and the non-radiative heat transfer coefficient, and present the ultimate lower bound of the temperature as well as the ultimate upper bound of the net radiative power density under those conditions.

## Theoretical model

**Figure 1:**
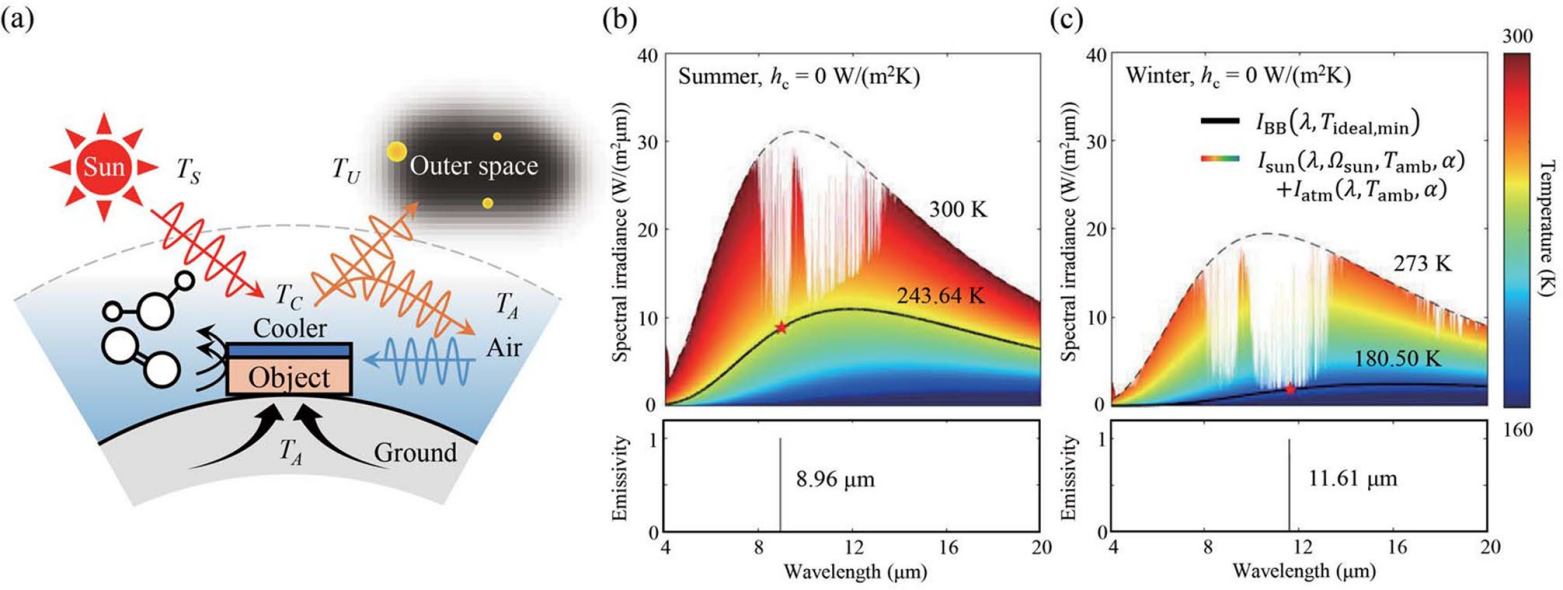
(**a**) Schematic of a radiative cooler and its surroundings. (**b**, **c**) Spectral irradiance of a blackbody at the steady state temperature of the ideal radiative cooler (black solid line) and that of the sun and the atmosphere combined (border line of the colored area) in (**b**) summer and (**c**) winter for *h*_c_ = 0 W/(m^2^K) at Daejeon city. Each color indicates the spectral irradiance of a blackbody at different temperatures (black dashed lines are for ambient temperature). The bottom panels show the ideal spectral emissivities.

Before we derive the ideal spectral emissivity, we provide our model and its assumptions on how a radiative cooler interacts with its surroundings. A radiative cooler that covers a target object is horizontally placed on the ground at sea level with no nearby obstacles so that the view angle of the sky is 2π steradian. The cooler and the object are in thermal equilibrium and thus have the same temperature (*T*). The cooler undergoes radiative heat exchange with the sun, the outer space, and the atmosphere. It also undergoes non-radiative, i.e., conductive and convective, heat exchange with the ambient air and the ground (Fig. 1a). The radiation through the atmosphere is subject to various conditions, such as season^16^, humidity^17^, and temperature variations along the altitude^18^. We denote these environmental variables as *α* and indicate the zenith angle of the sun as *Ω*_sun_. The ground is assumed to be at the same temperature as the ambient air (*T*_amb_). Then, the net cooling power density of the radiative cooler is given by

1 $${P}_{\mathrm{net}}\left({T}_{\mathrm{amb}},T,\alpha \right)={P}_{\mathrm{cooler}}\left(T\right)-{P}_{\mathrm{sun}}\left({\Omega }_{\mathrm{sun}}, {T}_{\mathrm{amb}},T,\alpha \right)-{P}_{\mathrm{space}}\left({T}_{\mathrm{amb}},T,\alpha \right)-{P}_{\mathrm{atm}}\left({T}_{\mathrm{amb}},T,\alpha \right)-{P}_{\mathrm{non}-\mathrm{rad}}\left({T}_{\mathrm{amb}},T\right),$$

where *P*_cooler_(*T*) is the radiant exitance of the cooler, *P*_sun_(*Ω*_sun_, *T*_amb_, *T*, *α*), *P*_space_(*T*_amb_, *T*, *α*), and *P*_atm_(*T*_amb_, *T*, *α*) are the absorbed irradiance on the cooler from the sun, the outer space, and the atmosphere, respectively, and *P*_non-rad_(*T*_amb_, *T*) is the absorbed non-radiative power density from the surroundings; these all have a unit of W/m^2^. *P*_space_(*T*_amb_, *T*, *α*) can be usually ignored because the cosmic background is much cooler than *T*_amb_ or *T*. The rest of the terms in Eq. (1) are expressed as

2a $${P}_{\mathrm{cooler}}\left(T\right)=\int \mathrm{d}\Omega \mathrm{cos}\theta \int \mathrm{d}\lambda {\stackrel{\sim }{I}}_{\mathrm{BB}}\left(\lambda ,T\right){\varepsilon }_{\mathrm{c}}\left(\lambda ,\Omega ,T\right),$$

2b $${P}_{\mathrm{sun}}\left({\Omega }_{\mathrm{sun}}, {T}_{\mathrm{amb}},T,\alpha \right)=\int \mathrm{d}\lambda {I}_{\mathrm{sun}}(\lambda ,{\Omega }_{\mathrm{sun}},{T}_{\mathrm{amb}},\alpha ){\varepsilon }_{\mathrm{c}}\left(\lambda ,{\Omega }_{\mathrm{sun}},T\right),$$

2c $${P}_{\mathrm{atm}}\left({T}_{\mathrm{amb}},T,\alpha \right)=\int \mathrm{d}\Omega \mathrm{cos}\theta \int \mathrm{d}\lambda {\stackrel{\sim }{I}}_{\mathrm{atm}}\left(\lambda ,\Omega ,{T}_{\mathrm{amb}},\alpha \right){\varepsilon }_{\mathrm{c}}\left(\lambda ,\Omega ,T\right),$$

2d $${P}_{\mathrm{non}-\mathrm{rad}}\left({T}_{\mathrm{amb}},T\right)={h}_{\mathrm{c}}({T}_{\mathrm{amb}}-T),$$

where $\int \mathrm{d}\Omega ={\int }_{0}^{2\uppi }\mathrm{d}\phi {\int }_{0}^{\uppi /2}\mathrm{d}\theta \mathrm{sin}\theta$ is the hemispherical integration and ${\stackrel{\sim }{I}}_{\mathrm{BB}}\left(\lambda ,T\right)=\frac{2{hc}^{2}}{{\lambda }^{5}}\frac{1}{\mathrm{exp}\left(hc/\lambda {k}_{\mathrm{B}}T\right)-1}$ is the spectral radiance of an ideal blackbody following Plank’s law (*h*, *c*, *k*_B_, and *λ* are the Plank constant, the velocity of light in vacuum, the Boltzmann constant, and wavelength, respectively). *ε*_c_(*λ*, *Ω*, *T*) represents the spectral and directional emissivity of the cooler. *I*_sun_(*λ*, *Ω*_sun_, *T*_amb_, *α*) is the spectral solar irradiance at a mid-latitude, sea-level location in the northern hemisphere when the sunlight is incident from angle *Ω*_sun_. In Eqs. (2b and 2c), the absorptivity of the cooler is replaced by its emissivity using Kirchhoff’s law. In Eq. (2d), the non-radiative absorption is expressed by an effective non-radiative heat transfer coefficient *h*_c_, which depends on environmental conditions, such as wind speeds, as well as on how well the cooler and the object are thermally insulated from the environment^19^. ${\stackrel{\sim }{I}}_{\mathrm{atm}}(\lambda ,\Omega ,{T}_{\mathrm{amb}}, \alpha )$ is the angle-dependent spectral radiance from the atmosphere, obtained by multiplying the black-body radiation spectrum with the atmospheric emissivity *ε*_atm_. The latter has been approximated as *ε*_atm_ (*λ*, *Ω*, *T*_amb_, *α*) = 1 − *t*(*λ*, *T*_amb_, *α*)^AM(*θ*)^^20^, where *t*(*λ*, *T*_amb_, *α*) is the atmospheric transmittance from the sea level toward the outer space in the zenith direction (data from MODTRAN 6^21^ with 2 nm spectral resolution) and AM(*θ*) accounts for larger attenuations at a non-zero zenith angle *θ*. In doing so, we applied a spherical shell model for AM(*θ*) (see Supplementary Methods and Fig. S1) instead of the flat-earth model of AM(*θ*) = 1/cos *θ*^20^.

## Results and discussion

Now, let us derive the ideal spectral emissivity of a radiative cooler at a specific temperature for maximum net cooling power density assuming it is isotropic at all angles. We first rearrange the net cooling power density in Eq. (1) into radiative and non-radiative parts as

3 $${P}_{\mathrm{net}}\left({\Omega }_{\mathrm{sun}},{T}_{\mathrm{amb}},T,\alpha \right)={P}_{\mathrm{rad}}\left({\Omega }_{\mathrm{sun}},{T}_{\mathrm{amb}},T,\alpha \right)-{P}_{\mathrm{non}-\mathrm{rad}}\left({T}_{\mathrm{amb}},T\right)=\int \mathrm{d}\lambda \left[{I}_{\mathrm{BB}}\left(\lambda ,T\right)-{I}_{\mathrm{sun}}(\lambda ,{\Omega }_{\mathrm{sun}},{T}_{\mathrm{amb}},\alpha )-{I}_{\mathrm{atm}}(\lambda ,{T}_{\mathrm{amb}}, \alpha )\right]{\varepsilon }_{\mathrm{c}}\left(\lambda ,T\right)-{h}_{\mathrm{c}}\left({T}_{\mathrm{amb}}-T\right),$$

where ${I}_{\mathrm{BB}}\left(\lambda ,T\right)=\int \mathrm{d}\Omega \mathrm{cos}\theta {\stackrel{\sim }{I}}_{\mathrm{BB}}\left(\lambda ,T\right)$ and ${I}_{\mathrm{atm}}\left(\lambda ,{T}_{\mathrm{amb}},\alpha \right)=\int \mathrm{d}\Omega \mathrm{cos}\theta {\stackrel{\sim }{I}}_{\mathrm{atm}}\left(\lambda ,\Omega ,{T}_{\mathrm{amb}},\alpha \right)$ are spectral irradiances and *P*_rad_(*Ω*_sun_, *T*_amb_, *T*, *α*) = *P*_cooler_(*T*) − *P*_sun_(*Ω*_sun_, *T*_amb_, *T*, *α*) – *P*_atm_(*T*_amb_, *T*, *α*) is the net radiative power density. We note that *ε*_c_(*λ*, *T*) works as a scaling factor for the net radiative heat exchange at each wavelength. For maximal *P*_net_(*Ω*_sun_, *T*_amb_, *T*, *α*) and *P*_rad_(*Ω*_sun_, *T*_amb_, *T*, *α*) at a given temperature, *ε*_c_(*λ*, *T*) can be suitably adjusted between 0 and 1 depending on the sign of the term *I*_rad,BB_ = *I*_BB_(*λ*, *T*) – *I*_sun_(*λ*, *Ω*_sun_, *T*_amb_, *α*) – *I*_atm_(*λ*, *T*_amb_, *α*). For wavelengths at which the sign is positive (i.e., net radiative emission occurring), *ε*_c_(*λ*, *T*) = 1 should be chosen to maximize the radiation whereas, for other wavelengths, *ε*_c_(*λ*, *T*) = 0 should be chosen to minimize absorption. This emissivity design rule is summarized as

4 $${\varepsilon }_{\mathrm{ideal}}\left(\lambda ;{\Omega }_{\mathrm{sun}},{T}_{\mathrm{amb}},T,\alpha \right)=\frac{1}{2}\left[1+\mathrm{sgn}\left({I}_{\mathrm{rad},\mathrm{BB}}\right)\right],$$

where $$\mathrm{sgn}\left(x\right)=\left\{\begin{array}{c} \begin{aligned}1\quad &\mathrm{if} \; x>0\\ -1 \quad & \mathrm{if} \; x<0\end{aligned}\end{array}\right.$$ is the sign function, whose value at *x* = 0 is undefined but does not affect the results in this situation.

We emphasize that *ε*_ideal_ is sensitively determined by the temperature of the cooler. This aspect was not fully investigated in previous studies. Two types of emissivity patterns were usually considered as optimal: a broadband emissivity pattern for above-ambient cooling and a rectangular (1 over 8–13 μm and 0 for other wavelengths) emissivity pattern for below-ambient cooling^6,7,14,22–26^. However, the actual spectral regions contributing to cooling and heating via radiation change dramatically as the temperature drops below the ambient temperature. Thus, emissivity spectra designed for a temperature range around ambient temperature are no longer an optimal solution at lower temperatures.

Another important fact revealed by Eq. (4) is that the temperatures achievable through radiative cooling have a fundamental lower bound. For *P*_rad_(*Ω*_sun_, *T*_amb_, *T*, *α*) to be positive, *I*_rad,BB_ must be positive at least for one wavelength; otherwise *ε*_ideal_ is zero at all wavelengths by Eq. (4) and net radiation will not occur. This imposes the ultimate lower bound, *T*_ideal,min_, for the temperature of a radiatively cooled object, where *T*_ideal,min_ is the temperature at which *I*_rad,BB_ is zero at one (or more) wavelengths and negative at all other wavelengths.

The temperature of the cooler in a steady state can be identified by finding *T* that satisfies *P*_net_(*Ω*_sun_, *T*_amb_, *T*, *α*) = 0 using the ideal emissivity specified by Eq. (4). First, we investigate the most extreme case of *h*_c_ = 0. Without non-radiative heat transfer, *P*_net_(*Ω*_sun_, *T*_amb_, *T*, *α*) for *ε*_ideal_ is always positive if *T* > *T*_ideal,min_. Thus, the ideal radiative cooler cools down until the temperature approaches *T*_ideal,min_. For example, at Daejeon city (36.35^*○*^ N in latitude), *T*_ideal,min_ is 243.6 K and 180.5 K at noon in summer and winter, respectively, as shown in Fig. 1b,c. This corresponds to 56.4 K and 92.5 K drops below ambient temperature (*T*_amb_ is assumed to be 300 K and 273 K in summer and winter, respectively). Owing to seasonal variations in atmospheric emissivity, the temperature drop is much larger in dry winter than in humid summer. As expected from Eq. (4), the ideal emissivity at these temperatures is a needle-like function centered at 8.96 μm in summer and 11.61 μm in winter; each single wavelength is the only one at which *I*_rad,BB_ is non-negative. Considering the lowest measured temperature in previous studies (approximately 243 K under vacuum conditions in winter)^5^, our results suggest that temperature can be further lowered by more than 50 K in principle. We note that this needle-like spectrum is a limiting case of *ε*_ideal_ at *T*_ideal,min_ and has zero net radiative power. It is still the ideal spectrum in terms of net radiative power since all other emitter design would have negative net radiative power at this temperature. It is also the ideal spectrum in terms of the steady-state temperature because no other design can reach this temperature at a steady state. Nonetheless, this design mainly serves as a theoretical limiting case and *ε*_ideal_ for higher temperatures is not a needle, with non-zero net radiative power (see Supplementary Fig. S2). It indicates that a temperature-sensitive *ε*_ideal_, if realized, would enable fast radiative cooling, due to its initial high net radiative power, down to a very low steady-state temperature (See Supplementary Methods, Figs. S3 and S4).

**Figure 2:**
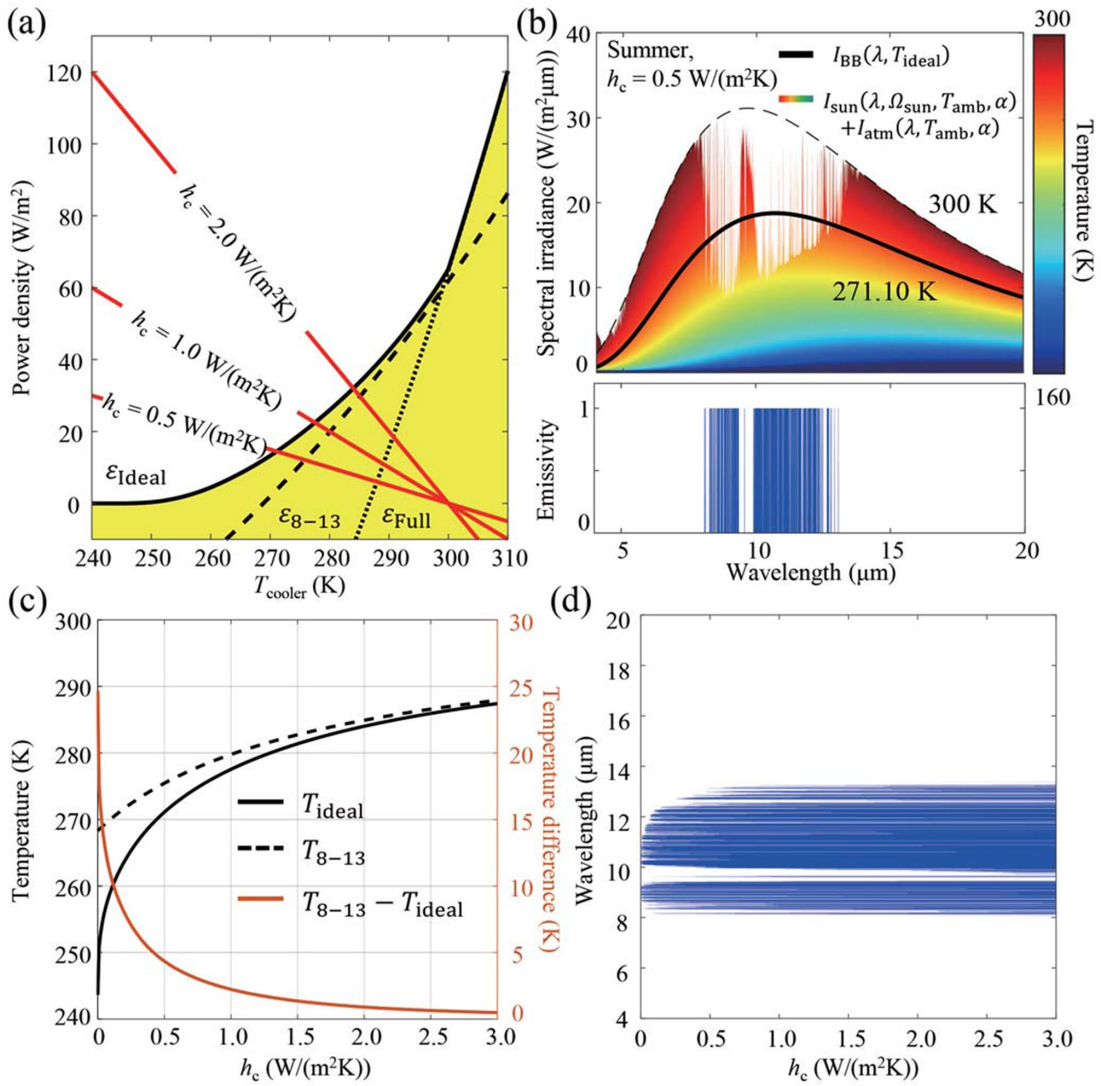
(**a**) Radiative (black) and non-radiative (red) power densities as a function of cooler temperature. (**b**) Spectral irradiance and spectral emissivity of the ideal radiative cooler for *h*_c_ = 0.5 W/(m^2^K). (**c**) The steady-state temperature as a function of *h*_c_. In (**a**, **c**), data for the ideal radiative cooler is plotted with black solid line, the 8–13 μm emitter with black dashed lines, and the broadband emitter with black dotted lines. (**d**) Ideal spectral emissivity as a function of *h*_c_.

In reality, the presence of conduction or convection (*h*_c_ ≠ 0) means that the steady-state temperature, *T*_ideal_(*h*_c_), of an ideal cooler designed for a given non-zero *h*_c_ value, is now a function of *h*_c_ and rises above *T*_ideal,min_ for non-zero *h*_c_; i.e., *T*_ideal,min_ defined above as the zero net radiative power temperature is the lower bound of *T*_ideal_(*h*_c_) and can be reached if and only if there is no non-radiative heat transfer (*T*_ideal,min_ = *T*_ideal_(*h*_c_ = 0)). The steady-state temperature of any radiative cooler with temperature-independent spectral emissivity can be easily found graphically because *P*_rad_ and *P*_non-rad_ are monotonically increasing and decreasing functions of *T*, respectively, and they cross each other at the steady-state temperature. For example, *P*_rad_'s for an 8–13 μm emitter (*ε*_8–13_, black dashed line) and a broadband emitter (*ε*_Full_, black dotted line) are plotted in Fig. 2a, under summer atmospheric conditions assuming solar irradiance corresponding to AM1.5 and an average solar zenith angle of 48.2°. The 8–13 μm emitter and the broadband emitter have a unity emissivity for wavelengths between 8 and 13 μm and for all wavelengths longer than 4 μm, respectively. Both hypothetical emitters have exactly zero emissivity for all other wavelengths. The red solid lines in Fig. 2a represent *P*_non-rad_ for several *h*_c_ values. As expected, the steady-state temperature for either emitter rises for higher *h*_c_ values.

In general, the net radiative power density of any radiative cooler cannot exceed the values indicated by the black solid line in Fig. 2a, representing the performance of an ideal radiative cooler at each cooler temperature as designed with Eq. (4). In previous studies, the 8–13 μm emitter was considered as an almost ideal radiative cooler for below-ambient cooling cases. However, it can be seen that the net radiative power density of this cooler (black dashed line in Fig. 2a) never touches the black solid line at any temperature. In other words, there is always a better spectral design than unit emissivity from 8 to 13 μm at any target temperature. For example, at 273.15 K, the net radiative power density of the 8–13 μm emitter is 8.21 W/m^2^, whereas that of the ideal cooler optimized for this temperature is more than two times larger at 16.84 W/m^2^. This is because the ideal cooler has much less radiation absorbed (48.06 W/m^2^ vs. 84.63 W/m^2^) even though its emission is also reduced (64.90 W/m^2^ vs. 92.84 W/m^2^) compared to the 8–13 μm emitter. Note that these results are independent of *h*_c_. For the particular case of *h*_c_ = 0.5 W/(m^2^K), the lower bound of the steady-state temperature, which is 271.10 K, is below the freezing temperature of water. The ideal cooler, which can reach this bound, has a highly selective spectral emissivity with many disjointed sets of wavelengths over which the emissivity is unity, as shown in Fig. 2b. Thus, the ideal spectral emissivity appears as a collection of several non-overlapping rectangular functions of unity amplitude added together. Thermal insulation on the level of *h*_c_ = 0.5 W/(m^2^K) can be achieved with a 7 cm-thick polystyrene foam at the back and an infrared-transmitting composite window at the front. Inside the previously accepted 8–13 μm transparency window, it is possible to identify several important wavelength ranges for which the emissivity must be minimized. One such wavelength range is around 9.5 μm, where there are multiple atmospheric absorption resonances due to O_3_ and other substances^23^. If these wavelengths are included in the emission band as done in previous designs, the steady-state temperature (275.45 K) rises above the freezing point of water.

At lower *h*_c_ values, the difference becomes more dramatic. *T*_ideal_ and *ε*_ideal_ are plotted for a range of *h*_c_ values in Fig. 2c,d. As *h*_c_ decreases, the difference between *T*_ideal_ and the steady-state temperature of the 8–13 μm emitter (*T*_8–13_) becomes larger, reaching 24.67 K at *h*_c_ = 0, as shown in Fig. 2c. The width of the wavelength ranges for which the emissivity should be unity diminishes as *h*_c_ is reduced and become highly selective, as illustrated in Fig. 2d. These results imply that, in a properly insulated system, it is possible to achieve a much lower steady-state temperature than *T*_8–13_ if the spectral emissivity is optimally designed to benefit from the lower *h*_c_.

**Figure 3:**
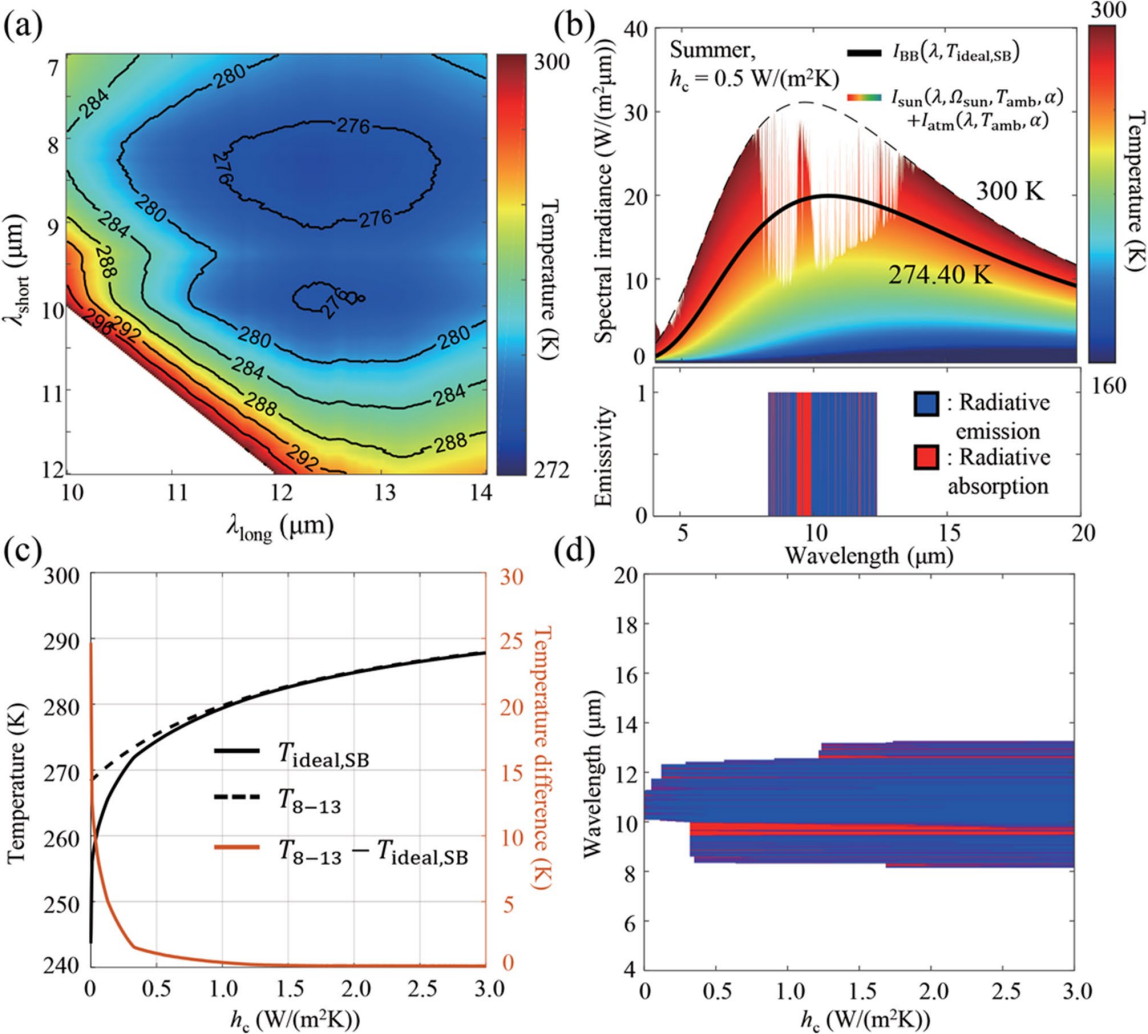
(**a**) The steady-state temperature of a single-band emitter for various *λ*_short_ and *λ*_long_ combinations. (**b**) Spectral irradiances (top panel) and the spectral emissivity of an ideal single-band emitter for *h*_c_ = 0.5 W/(m^2^K) (bottom panel). The blue and red areas in the bottom panel indicate the spectral regions of emission and absorption via radiation, respectively. (**c**) The steady-state temperature of the ideal single-band emitter (black solid line) and the 8–13 μm emitter (black dashed line), and the difference between them (red line), as a function of *h*_c_. (**d**) Ideal single-band spectral emissivity as a function of *h*_c_.

In practice, however, it might be challenging to realize such a highly selective spectrum. Thus, we also consider a simper, single-band emitter with unit emissivity over a single wavelength range from *λ*_short_ to *λ*_long_ and optimize *λ*_short_ and *λ*_long_ for best performance. For *h*_c_ = 0.5 W/(m^2^K), the steady-state temperature is shown in Fig. 3a for different combinations of *λ*_short_ and *λ*_long_. Among the various potential designs, the optimal design is *λ*_short_ = 8.30 μm and *λ*_long_ = 12.38 μm, with a corresponding steady-state temperature of 274.40 K. Whereas the ideal, multi-band emitters depicted in Fig. 2 have non-negative net radiative emission at all wavelengths, single-band emitters have net radiative absorption at some wavelength regions, as shown in Fig. 3b with a red color. Nonetheless, an optimally designed single-band emitter can exhibit a considerably lower steady-state temperature (*T*_ideal,SB_) than *T*_8–13_ for highly insulated systems (Fig. 3c). In particular, at the perfect insulation limit, *T*_ideal,SB_ and *T*_8–13_ converge to 243.64 K and 268.31 K, respectively, exhibiting a difference of 24.67 K. Even in a more realistic case of *h*_c_ = 0.13 W/(m^2^K), *T*_ideal,SB_ (265.60 K) is 5 K lower than *T*_8–13_ and the corresponding emission band is from 9.98 to 12.26 μm. Figure 3d illustrates the optimal emission band for a wide range of *h*_c_ values. At high *h*_c_ values, the ideal emission band for a single-band radiative cooler is similar to previous designs of 8–13 μm. However, at low *h*_c_ values, the optimal emission band narrows down considerably. In particular, it can be seen from Fig. 3c,d that it is better to abandon the wavelength range from 8 to 10 μm if the target steady-state temperature is lower than the freezing point of water. Of course, if dual or multi-band designs are permissible, a part of this wavelength range can be used for radiation to decrease the steady-state temperature further or to increase the net radiative power density.

For direct comparison, we present Table 1 that shows the steady-state temperatures of ideal and non-ideal coolers for various *h*_c_ conditions. The spectrally-selective and single-band based ideal coolers outperform other non-ideal coolers by many degrees for small *h*_c_. Even for *h*_c_ = 2 W/(m^2^K) that is practically realizable even without vacuum sealing^26^, the ideal cooler shows noticeable advantage.

**Table 1:** Steady-state temperature of spectral emissivities depending on *h*_c_.

*h*_c_ (W/(m^2^K))	Steady-state temperature (K)				
	ɛ_ideal_	*ɛ*_8–13_	*ɛ*_full_	*ɛ*_ideal,SB_	*ɛ*_Ref_^3^
0	243.6	268.3 (↑24.7)	286.6 (↑43.0)	243.6 (↑0.0)	279.0 (↑35.4)
0.5	271.1	275.5 (↑4.4)	288.0 (↑16.9)	274.4 (↑3.3)	283.8 (↑12.7)
1.0	277.5	279.8 (↑2.3)	289.0 (↑11.5)	279.4 (↑1.9)	286.7 (↑9.2)
2.0	284.0	284.9 (↑0.9)	290.7 (↑6.7)	284.8 (↑0.8)	290.2 (↑6.2)

ɛ_Ref._^3^ is the spectral emissivity from Ref.^3^. The number in parentheses presents temperature increase of other emissivities compared to ɛ_ideal_.

In conclusion, we presented a systematic method to calculate the ultimate lower bound of a radiatively cooled object’s steady-state temperature as well as the ultimate upper bound of net radiative power density at a given cooler temperature under general environmental conditions with an arbitrary effective non-radiative heat transfer coefficient. We also derived the ideal spectral emissivities that can reach such bounds. Unlike the often-adopted contiguous emission window of 8–13 μm used in previous radiative coolers, the ideal radiative cooler exhibits unity emissivity over disjointed sets of wavelengths. We also investigated the ideal emission band for a single-band emitter and found that the optimal band narrows down considerably at lower temperatures. The proposed scheme may serve as a basic guideline for designing the emissive properties of extreme radiative coolers as well as for estimating the amount of thermal insulation required for them.

## Supplementary information

Supplementary Information.
