# Supplementary material for: Ideal spectral emissivity for radiative cooling of earthbound objects
Source: Sci Rep. 2020 Aug 3;10:13038. doi: 10.1038/s41598-020-70105-y (PMC7400651; doi:10.1038/s41598-020-70105-y)
Supplement: Supplementary file 1 — Supplementary Information. [file 41598_2020_70105_MOESM1_ESM.pdf]

Supplementary material

Ideal spectral emissivity for radiative cooling of earthbound objects

Suwan Jeon & Jonghwa Shin*

Department of Materials Science and Engineering, Korea Advanced Institute of Science and Technology, Daejeon 34141, Republic of Korea

*qubit@kaist.ac.kr

Supplementary Methods

Spherical shell model

As electromagnetic wave transmitting through the atmosphere, it is scattered and absorbed by air molecules. The level of attenuation depends on atmospheric thickness along the line-of-sight, which can be represented by the angle of sight. In order to evaluate cooling performance of radiative coolers, which normally emit thermal radiation over all directions, angular properties of atmospheric transmittance must be precisely described. From Beer-Lambert law, the transmittance through lossy medium, i.e., the atmosphere, at the zenith angle θ can be expressed as $T(\theta) = 10^{-A(\theta)}$. The angle-dependent attenuation coefficient $A(\theta)$ can be approximated as $A(\theta) = \mathrm{A_0}\cdot h(\theta)/\mathrm{h_0}$, where $\mathrm{A_0}$ is the attenuation constant, $h(\theta)$ is the angle-dependent thickness of the atmosphere, and $\mathrm{h_0}$ is the atmospheric thickness at the zenith direction. Then, the angle-dependent transmittance through the atmosphere can be arranged as

$$T(\theta) = 10^{-A(\theta)} = (10^{-\mathrm{A_0}})^{h(\theta)/\mathrm{h_0}} = (\mathrm{T_0})^{\mathrm{AM}(\theta)} \tag{S1}$$

where $\mathrm{T_0}$ is the atmospheric transmittance at the zenith direction (denoted as $t(\lambda, T_{\mathrm{amb}}, \alpha)$ in the manuscript to express spectral and environmental effects) and $\mathrm{AM}(\theta) = h(\theta)/\mathrm{h_0}$ is the air mass coefficient at the zenith angle θ. In flat earth model, mostly used in previous studies, the air mass coefficient is $\mathrm{AM}(\theta) = 1/\cos\theta$. Although it might be a simple model expressed only with zenith angle, the air mass coefficient diverges at large zenith angle, close to 90°. On the other hand, spherical shell model can represent atmospheric thickness even at large zenith angle by assuming that the atmosphere surrounds the earth just like a spherical shell (Fig. S1(a)). In this paper, we applied geometric parameters as $\mathrm{h_0}$ = 99 km and R = 6400 km. Figure S1(b) illustrates that spherical shell model is reliable over 70° contrary to flat earth model.

Cooling speed

The cooling speed via radiation can be obtained by solving the differential equation [S1] as,

$$C_{cooler}\frac{dT}{dt} = P_{net}(T_{amb}, T, \alpha) \quad \text{(R2)}$$

where $C_{\text{cooler}} = \rho \times c \times l$ is heat capacity density (ρ, *c*, and *l* are density, specific heat capacity, and thickness of the cooler, respectively) with a unit of kJ/(m^{2}k) and P_{net} is net cooling power density of the cooler. For radiative cooling, we concern the power per unit area, so we can quantify the amount of cooler with heat capacity density, and C_{cooler} is directly determined by thickness of the cooler when the material (or material combinations) of cooler is specified. For instance, glass and water with 1 mm thickness correspond to C_{cooler} = 1.85 kJ/(m^{2}k) and 4.19 kJ/(m^{2}k), respectively.

Without non-radiative effect as $h_c = 0$, temperature drop of the cooler with various C_{cooler} over time is illustrated in Supplementary Fig. S3. As expected, our ideal emitter cools faster and lower than 8–13 µm emitter and 4–20 µm emitter. To cool down to freezing point of water (273.15 K) within 10, 30, and 60 min, maximally allowable C_{cooler} of the ideal cooler are 0.72, 2.17, and 4.35 kJ/(m^{2}k), respectively. This indicates that the water of 1 mm thickness can be cooled to the freezing point within 1 hour. However, 4–20 µm emitter never reach the freezing point of water and 8–13 µm emitter also cannot cool down below 268.31 K. Whereas, the ideal emitter with C_{cooler} = 3.05 kJ/(m^{2}k) can cool to the temperature limit of 8–13 µm (268.31 K) in 1 hour. Under non-radiative effect, incoming heat may slow down the cooling speed but the ideal emitter still outperforms other emitters regarding cooling speed and cooled temperature, as shown in Supplementary Fig. S4. Especially, the ideal emitter cools below temperature limit of 8–13 µm emitter within 1 hour, when its heat capacity density is smaller than 2 kJ/(m^{2}k). The ideal cooler also cools below freezing point of water within 1 hour when its C_{cooler} is smaller than 2.4 kJ/(m^{2}k) that corresponds to 1.3 mm thickness of glass, whereas 8–13 µm emitter allows C_{cooler} below 0.01 kJ/(m^{2}k) that corresponds to 0.005 mm thickness of glass.

References

[S1] Ono, Masashi, et al. "Self-adaptive radiative cooling based on phase change materials." *Optics express* 26, A777-A787 (2018).

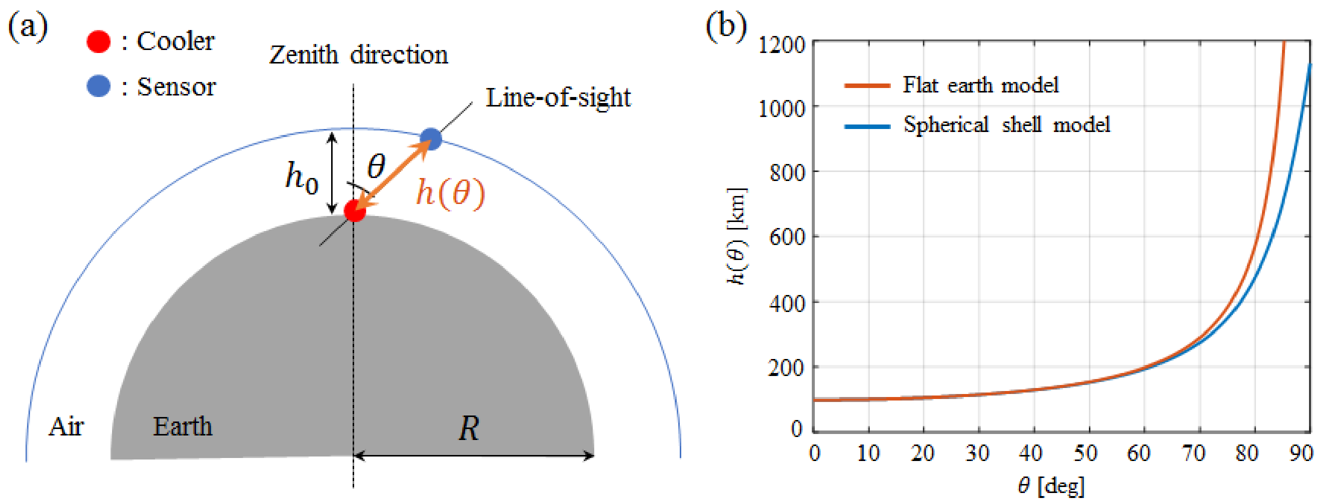

Supplementary Figure S1. (a) Schematic of spherical shell model and (b) atmospheric thickness as a function of the zenith angle for flat earth model and spherical shell model.

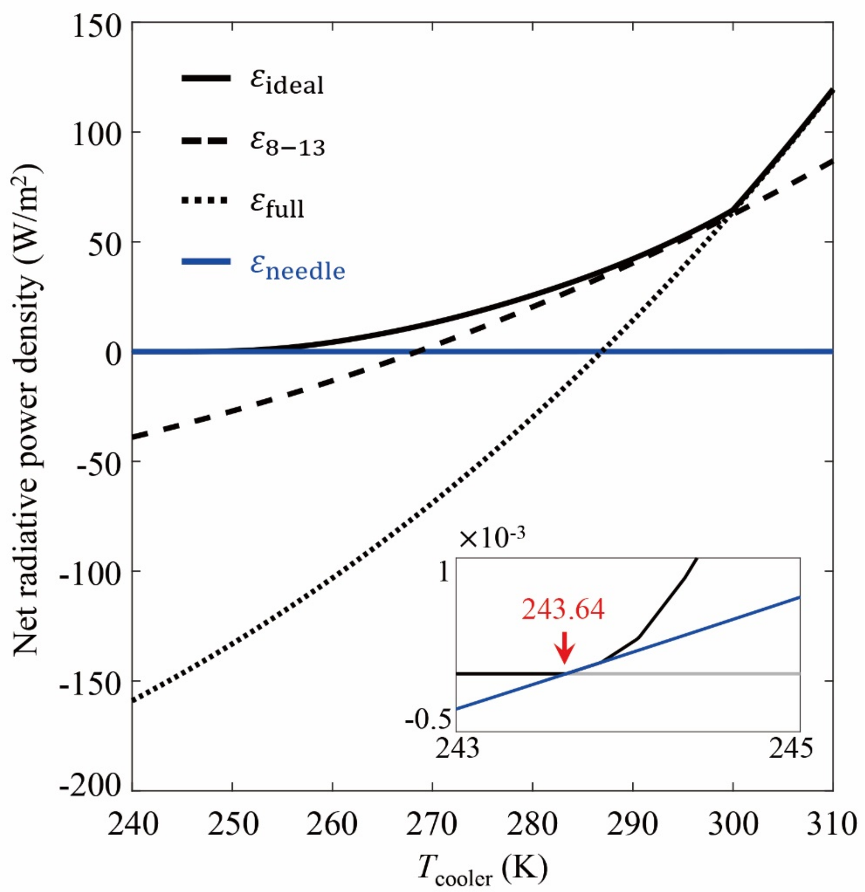

Supplementary Figure S2. Net radiative cooling power density P_{rad} as a function of temperature for various emissivities. The red arrow in inset indicates the ultimate lower bound of cooling temperature $T_{ideal,min}$. The gray line in inset is a reference line for zero net radiative power density.

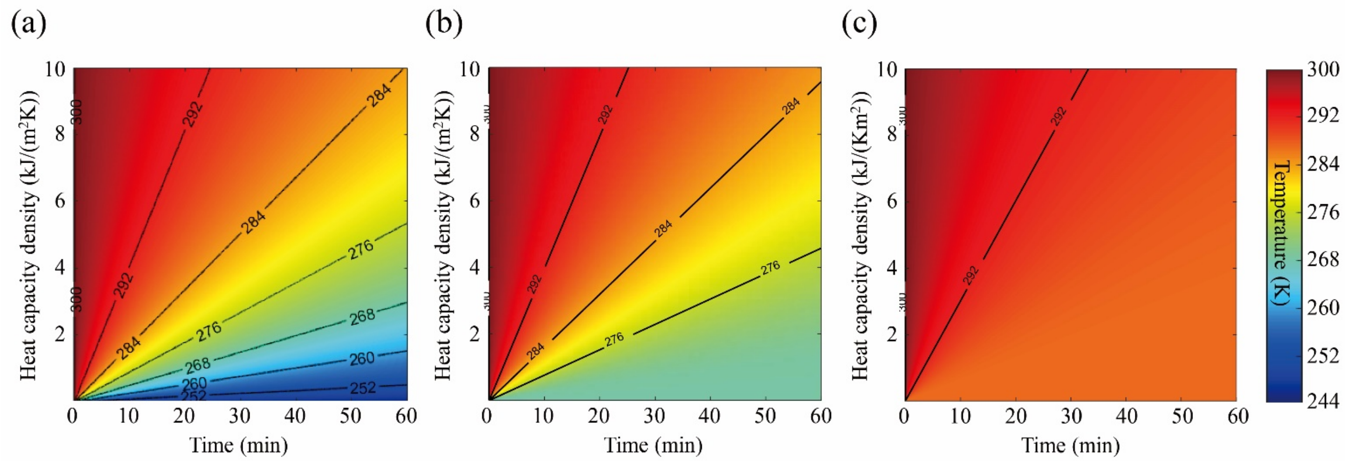

Supplementary Figure S3. Temperature drop over time for (a) ideal emitter, (b) 8–13 µm emitter, and (c) 4-20 µm emitter, along with different heat capacity density. In this plot, h_c = 0 W/(m^{2}k).

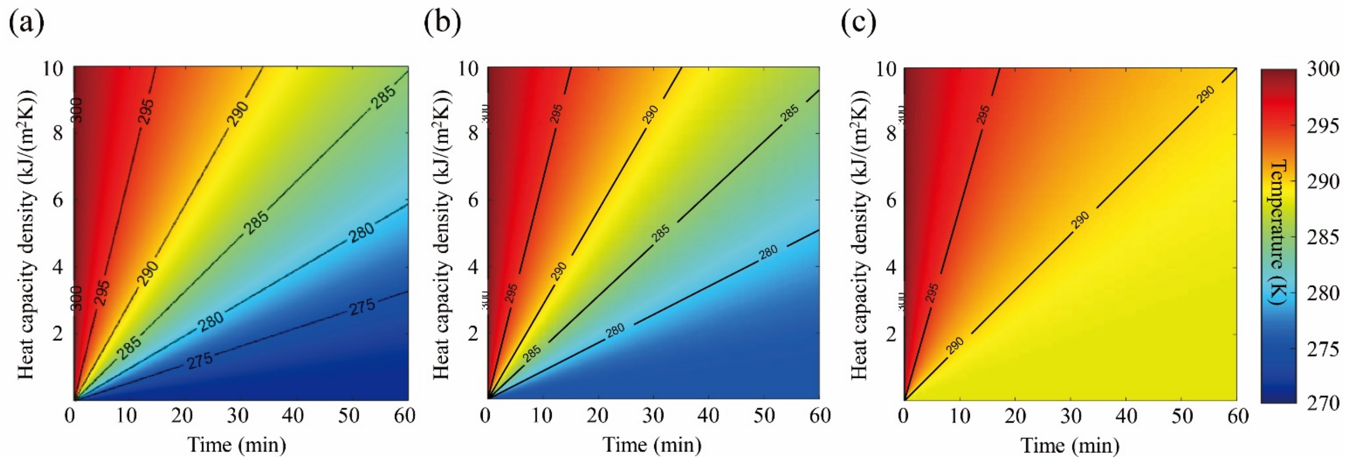

Supplementary Figure S4. Temperature drop over time for (a) ideal emitter, (b) 8–13 µm emitter, and (c) 4-20 µm emitter, along with different heat capacity density. In this plot, h_c = 0.5 W/(m^{2}k).
